# A microRNA–microRNA crosstalk network inferred from genome-wide single nucleotide polymorphism variants in natural populations of *Arabidopsis thaliana*

**DOI:** 10.3389/fpls.2022.958520

**Published:** 2022-08-26

**Authors:** Xiaomei Wu, Xuewen Wang, Wei Chen, Xunyan Liu, Yibin Lin, Fengfeng Wang, Lulu Liu, Yijun Meng

**Affiliations:** ^1^College of Life and Environmental Sciences, Hangzhou Normal University, Hangzhou, China; ^2^Department of Genetics, University of Georgia, Athens, GA, United States; ^3^College of Life Sciences, Zhejiang University, Hangzhou, China; ^4^College of Life Sciences and Oceanography, Shenzhen University, Shenzhen, China

**Keywords:** miRNA–miRNA crosstalk, *Arabidopsis thaliana* ecotypes, SNP, regulation fate profile, climatic variables, stress response

## Abstract

To adapt to variable natural conditions, plants have evolved several strategies to respond to different environmental stresses. MicroRNA (miRNA)-mediated gene regulation is one of such strategies. Variants, e.g., single nucleotide polymorphisms (SNPs) within the mature miRNAs or their target sites may cause the alteration of regulatory networks and serious phenotype changes. In this study, we proposed a novel approach to construct a miRNA–miRNA crosstalk network in *Arabidopsis thaliana* based on the notion that two cooperative miRNAs toward common targets are under a strong pressure to be inherited together across ecotypes. By performing a genome-wide scan of the SNPs within the mature miRNAs and their target sites, we defined a “regulation fate profile” to describe a miRNA–target regulation being static (kept) or dynamic (gained or lost) across 1,135 ecotypes compared with the reference genome of Col-0. The cooperative miRNA pairs were identified by estimating the similarity of their regulation fate profiles toward the common targets. The reliability of the cooperative miRNA pairs was supported by solid expressional correlation, high PPImiRFS scores, and similar stress responses. Different combinations of static and dynamic miRNA–target regulations account for the cooperative miRNA pairs acting on various biological characteristics of miRNA conservation, expression, homology, and stress response. Interestingly, the targets that are co-regulated dynamically by both cooperative miRNAs are more likely to be responsive to stress. Hence, stress-related genes probably bear selective pressures in a certain group of ecotypes, in which miRNA regulations on the stress genes reprogram. Finally, three case studies showed that reprogramming miRNA–miRNA crosstalk toward the targets in specific ecotypes was associated with these ecotypes’ climatic variables and geographical locations. Our study highlights the potential of miRNA–miRNA crosstalk as a genetic basis underlying environmental adaptation in natural populations.

## Introduction

Plant populations growing under natural conditions are exposed to different environmental stresses in the form of a combination of various climatic, edaphic, and other abiotic factors (Tripathi et al., 2019). Adaptation to local conditions has been shown experimentally in many organisms (Hereford, 2009). Moreover, the capacity to respond to complex environmental stresses is likely to vary among species in their degree of phenotypic plasticity and their potential for genetic adaptation (Hoffmann and Sgro, 2011). Mean survival and lifetime fruit production differed markedly across *Arabidopsis thaliana* ecotypes within the same planting site, suggesting heritable variation among source populations in fecundity and viability (Fournier-Level et al., 2011). Growing season length, which depends on photoperiod, temperature, and rainfall patterns, drastically changes even over short geographic distances (Exposito-Alonso, 2020). Single nucleotide polymorphisms (SNPs) are important sequence variations for the diversity among individuals and are ubiquitously present in most organisms (Arai-Kichise et al., 2011). By investigating SNPs linked to loci experiencing real-time selection in different natural environments, Fournier-Level and his colleagues (2011) found that the genetic basis of fitness in *A. thaliana* differs dramatically across sites (Fournier-Level et al., 2011). Moreover, the maintenance of sufficient standing genetic variation is essential for adaptation to rapid climate change (Fournier-Level et al., 2011, 2016).

To adapt to different environmental conditions, plants have evolved several strategies to cope with varying biotic and abiotic stresses. One of such strategies is the microRNA (miRNA)-mediated post-transcriptional gene regulation. The plant miRNAs are a class of non-coding small RNAs with ~21 nucleotides in length. They are processed from the hairpin-structured precursors by DCL1 in the nucleus and incorporated into the AGO1 complex to target the mRNA (s) in the cytoplasm (Dalmadi et al., 2019). Growing evidence shows that miRNAs are key post-transcriptional regulators of gene expression and play crucial roles in diverse biological processes to cope with varying environmental stresses (Chiou, 2007; Sunkar et al., 2012; Ferdous et al., 2015; Basso et al., 2019). SNPs within the mature miRNAs and their target binding sites may function as the regulatory genetic codes influencing the miRNA–target pairs, thus further causing the physiological or phenotypic changes (Gong et al., 2012; Liu et al., 2013, 2016, 2021). In many cases, one miRNA can target more than one gene, suggesting the function complexity of miRNAs. One gene can also be regulated by more than one miRNA, thereby indicating cooperative control among multiple miRNAs (Xu et al., 2011). At present, the combinatorial nature of miRNA regulation has been detected by using several experimental approaches and is widely accepted in multiple species (Vella et al., 2004; Curaba et al., 2014; Samad et al., 2017; Lai et al., 2019). Therefore, studying the cross among miRNAs, rather than the individual miRNA–target regulation, can greatly improve our understanding of the potential functional effects of the complex interplay between miRNAs.

Different bioinformatics pipelines have been developed to infer synergistic regulation among miRNAs based on the genomic similarity, co-regulation, co-functionality, co-expression, and SNP cooperation of miRNA pairs (Xu et al., 2017). In genomic similarity, Xu et al. (2013) demonstrated that functional synergetic miRNA pairs exhibit high seed sequence similarity. Chen et al. (2014) analyzed the impacts of the three-dimensional architecture of chromatin on the transcriptional regulation of miRNAs. They indicated the existence of spatial miRNA–miRNA chromatin interacting networks by assembling miRNA pairs that interact with each other at the chromatin level. The functional roles of miRNAs can be deciphered through their target genes (Fan and Kurgan, 2015). Thus, many methods have been developed to detect cooperation between miRNAs by identifying miRNA pairs that co-regulate at least one target in a statistical framework, such as mirBridge (Tsang et al., 2010), GeneSet2miRNA (Antonov et al., 2009), and miRror2.0 (Balaga et al., 2012; Friedman et al., 2014). Another common assumption is that the genes regulated by multiple miRNAs should be functionally associated in terms of the co-regulation of targets. Therefore, many methods have been proposed to detect miRNA pairs with similar functions based on genes regulated. To address the issue, Gene Ontology (GO) annotations, pathways, and protein–protein interaction networks were used to evaluate the functional similarity among the genes regulated by miRNA groups and have been applied in human (Xu et al., 2011; Sun et al., 2013) and several plants, including Arabidopsis (Meng et al., 2015), rice (Banerjee and Mal, 2020), and soybean (Xu et al., 2014). Increasing evidence suggests that miRNA–mRNA regulation is context-specific. Thus, the expression relationships between miRNA and mRNA were examined to identify the miRNA–mRNA regulations, before constructing the context-specific miRNA–miRNA crosstalk network using the methods mentioned above. Furthermore, Zhang et al. (2019) simulated multiple knockouts of miRNAs using gene expression data and apply causal inference methods to find synergistic miRNA pairs. They found that most of synergistic miRNA–miRNA pairs tend to be co-expressed. Co-expression of miRNA pairs has been used to filter the identified miRNA–miRNA pairs (Song et al., 2015; Shao et al., 2019). Alternatively, Hua et al. (2014) constructed a coronary artery disease-related miRNA–miRNA synergistic network by combining miRNA expression profiling data with genome-wide SNP genotype data. The method is based on the hypothesis that SNPs in the target binding sites of two cooperative miRNA pairs are correlated among the human populations. Firstly, the differentially expressed miRNAs are identified using coronary artery disease-related miRNA expression data. Then, the miRNASNP tool (Gong et al., 2012) is used to extract the pairs of differentially expressed miRNAs and mutant-type target transcripts (the transcripts with 3′-UTR SNPs) that are gained in the human populations compared with the reference human genome. Finally, logistic regression is used to detect the significant interactions between 3′-UTR target SNPs of differentially expressed miRNAs among the human populations. In this way, the miRNA–miRNA cooperation pairs are identified based on the associated SNP pairs (Hua et al., 2014).

The study by Hua et al. (2014) provides evidence that miRNA regulation could be reprogrammed in different human genomes. Nevertheless, the method by Hua et al. ignored two critical aspects of information embedded in the miRNA–target regulatory process across different genomes, which would greatly improve the performance: (1) In addition to the variations in the mRNA 3′UTR, variations in the miRNAs are also important genetic basis for the alteration of miRNA–target pairs in different genomes. (2) In addition to the newly gained miRNA–target pairs, the miRNA–target pairs in the reference genome could be completely dropped or still kept in other genomes (Gong et al., 2012; Liu et al., 2021).

*Arabidopsis thaliana* is an established plant model for deciphering genetic basis and ecological adaptations owing to its world-wide distribution and the availability of genome-wide SNP data (Hancock et al., 2011; The 1001 Genomes Consortium, 2016). In this study, we proposed a novel approach to identify cooperative miRNA pairs in *A. thaliana* based on the notion that if two miRNAs are synergistic to regulate the same target, there will be a strong pressure on the two miRNAs to follow the same mode of miRNA regulation reprogramming across ecotypes, that is the regulation pairs are likely to coexist across ecotypes. The constructed miRNA–miRNA crosstalk network is of high quality as the synergistic miRNA pairs show co-expression and are enriched with miRNA pairs in response to identical types of stress. We also investigated the biological significance of miRNA–miRNA pairs classified by the crosstalk type toward the common targets, both regulation pairs being static (“SS”), both being dynamic (“DD”), and one being static while the other being dynamic (“SD”). MiRNA–miRNA pairs with various crosstalk types present the different biological significance in terms of miRNA conservation, expression, homology, and regulatory SNP distribution. Finally, we investigated the specific ecotypes in which the regulations toward each transcript are gained or lost, and proposed that these ecotypes are exposed to similar climatic conditions. This is the first report studying cooperative control among miRNAs at the level of natural populations of *A. thaliana*. This study may provide further insight into the potential functional effects of cooperative miRNAs in the adaptation of populations under different environmental conditions.

## Materials and methods

### miRNA–target regulations in the reference genome and across ecotypes

A three-step pipeline was used to generate a population-level miRNA–target regulation network. Firstly, *A. thaliana* Columbia-0 (Col-0) reference genome sequence (TAIR10 release) and genome annotation (Araport11 with the release as June 2016) were derived from the TAIR database.^1^ Four hundred twenty-eight mature miRNAs were derived from the miRBase database (release 22; Griffiths-Jones, 2004; Kozomara et al., 2019). The *A. thaliana* variant data set, which contains SNPs and short insertion and deletion variants (indels) of 1,135 *A. thaliana* ecotypes, was derived from the 1001 Genomes ftp^2^ (The 1001 Genomes Consortium, 2016). These variants are based on intersection of the SHORE and GATK pipeline. The variants at both population (1001genomes_snp-short-indel_only_ACGTN.vcf.gz) and individual ecotype (folder intersection_snp_short_indel_vcf) levels were downloaded. The biallelic variants with one allele in only one ecotype (singletons), accounting for 67% total variants, were removed (Supplementary Table S1). The average variant density is 64.16 variants per kb.

Secondly, miRNA target sites in the *A. thaliana* reference genome and 1,135 ecotype pseudogenomes were predicted. To prevent the bias produced by an individual prediction tool and obtain more satisfactory results, two plant miRNA target identification tools, TargetFinder (Fahlgren et al., 2007) and PsRobot (Wu et al., 2012) with the default parameters were applied. Such a strategy has also been used in several previously studies (Gong et al., 2012; Liu et al., 2013; Meng et al., 2015). In the reference genome, wild-type mature miRNAs and wild-type transcripts were input to the tools. In each ecotype, miRNA and transcript sequences were divided into wild type (lacking genetic diversity) and corresponding mutant type (presenting genetic diversity). Indels occurring in protein-coding sequence may cause frameshifts (changing the reading frame) with drastic changes to protein sequence and nearly always inactivate proteins. Thus, for each ecotype, the miRNAs and transcripts that contain any short indels were discarded. There are 0%–0.9% of miRNAs and 0.3%–24.8% of transcripts containing short indels. The kept miRNAs and transcripts only contain SNPs. New sequences of mutant-type miRNAs and mutant-type transcripts were retrieved. miRNA target sites were re-predicted from wild- and mutant-type miRNAs as well as wild- and mutant-type transcripts. Then, we defined lost, gained and kept miRNA–target pairs compared with the reference *A. thaliana* genome. If one miRNA–target transcript pair was found by both tools in the reference genome, but neither by TargetFinder or PsRobot in a ecotype, we defined that the miRNA loses the target, because of the presence of SNP(s) in the miRNA or target transcript (Formula 1). In contrast, if one miRNA–target regulation was predicted by both tools in the ecotype, but neither by TargetFinder or PsRobot in the reference genome, we defined that the miRNA gains the corresponding target transcript in this ecotype (Formula 2). Kept miRNA–target regulations were defined as predicted by both tools in the reference genome and in the ecotype genome (Formula 3).


(1) $$\mathrm{Lost regulations}=\left(T_{ref}\cap P_{ref}\right)-\left(T_{eco}\cup P_{eco}\right)$$

(2) $$\mathrm{Gained regulations}=\left(T_{eco}\cap P_{eco}\right)-\left(T_{ref}\cup P_{ref}\right)$$

(3) $$\mathrm{Kept regulations}=\left(T_{ref}\cap P_{ref}\right)+\left(T_{eco}\cap P_{eco}\right)$$

where *T*_ref_ and *T*_eco_ are the miRNA–target transcript pairs predicted by TargetFinder in the reference and ecotype genomes, respectively. *P*_ref_ and *P*_eco_ are the miRNA–target transcript pairs predicted by PsRobot in the reference and ecotype genomes, respectively.

Thirdly, all miRNA–target regulation relations in 1,135 ecotypes were combined at the population level. Each edge between miRNA and target transcript has a property of fate. A miRNA–target pair which is present in the reference genome, may be kept in all ecotypes (the general fate = “K” means fate = “k” in each ecotype), may be kept (fate = “k”) in some ecotypes but lost (fate = “l” in at least one ecotype) in some others (the general fate = “KL”), or may be lost in all ecotypes (not found in our data). A miRNA–target pair which is not present in the reference genome may be gained (fate = “g”) in at least one ecotype (the general fate = “G”). The miRNA–target pair with the general fate as KL or G represents that it is in a dynamic state of being dropped or gained in some ecotypes and is called the “dynamic” regulation, whereas the miRNA–target pair with the general fate as K is called the “static” regulation.

### Construction of miRNA–miRNA crosstalk network

**Figure 1:**
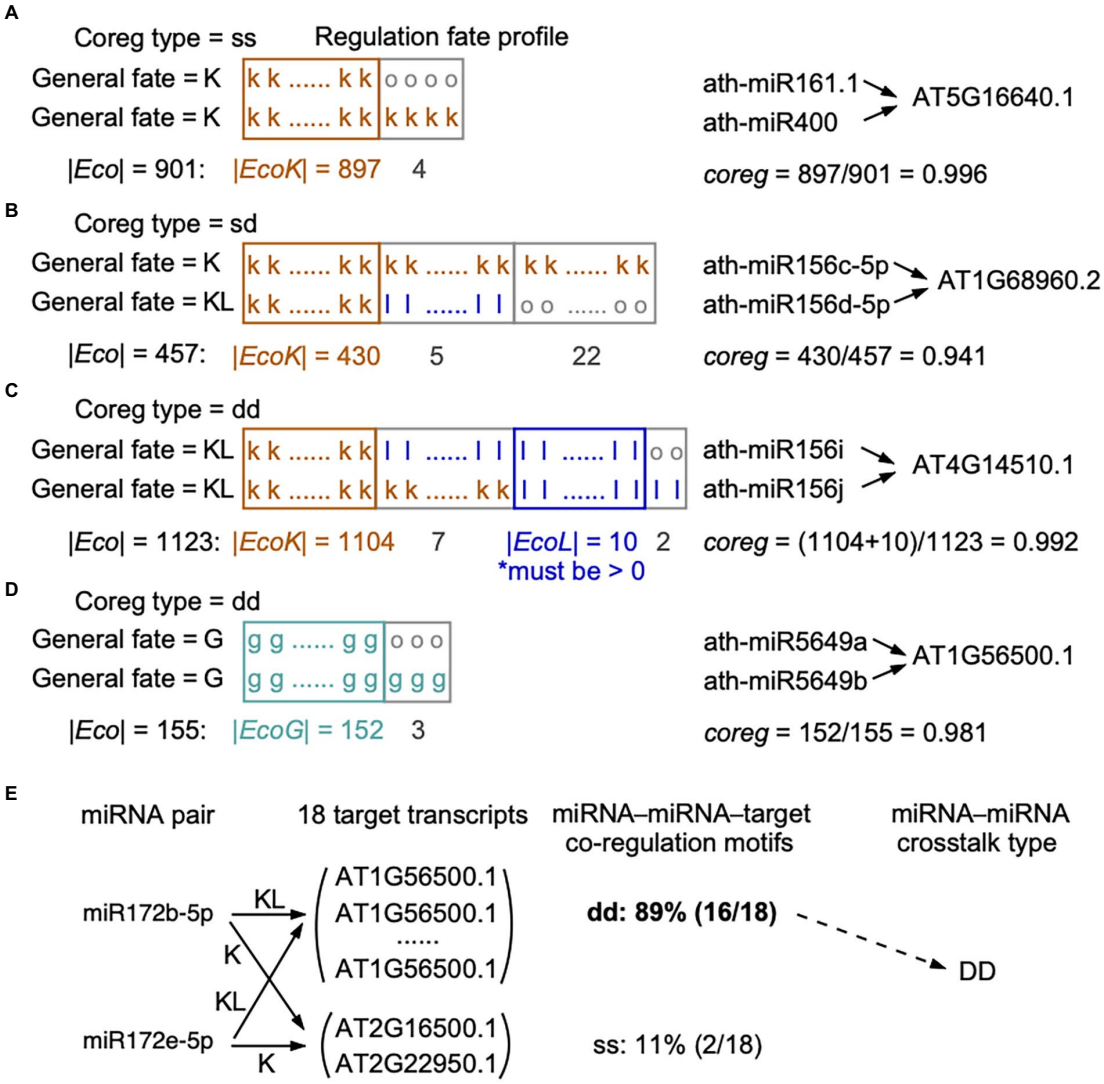
Calculation of co-regulation (*coreg*) score based on the regulation fate profiles. **(A-D)** Four different conditions for calculating coreg score were presented. When two miRNAs co-regulate a certain target in the reference genome, loss events in some ecotypes may happen **(A)** to none regulatory pairs (their general fates as “K” and “K”), **(B)** to only one regulatory pair (“K” and “KL”), or **(C)** to both regulatory pairs (“KL” and “KL”). **(D)** Two miRNAs may gain a common target in some ecotypes but the two regulatory pairs are not present in the reference genome (the general fates as “G” and “G”). In regulation fate profiles, the fate in each ecotype is “k,” “l,” “g,” or “o”. A miRNA–target regulation marked with the fate as “o” in an ecotype means this regulation was detected by only one miRNA target prediction tool in this ecotype. We required that in **(C)**, *EcoL*, the set of ecotypes, in which the two regulatory pairs are lost, should not be empty, to make sure the two regulatory pairs were dropped together in at least one ecotype. The co-regulation type of a miRNA–miRNA–target motif (“ss”: static-static, “dd”: dynamic-dynamic, and “sd”: static-dynamic) was defined according to the general fates of the two regulatory pairs (see Materials and methods). **(E)** An example showing the definition of the crosstalk type for a cooperative miRNA pair. Ath-miR172b-5p and ath-miR172e-5p target 18 common transcripts, 11% (2 out of 18) of transcripts are targeted with the co-regulation type of ss and 89% (16 out of 18) of transcripts are targeted with the co-regulation type of dd. We considered that ath-miR172b-5p and ath-miR172e-5p cooperatively interact with DD as the crosstalk type, because more than half of the common targets are co-regulated with the co-regulation type of dd (see Materials and methods).

The notion for our method is that a cooperative miRNA–miRNA pair tends to regulate the same target transcript and moreover, the two regulatory pairs between miRNA and target coexist in natural populations. In other words, if two miRNAs cooperatively regulate a target, there will be a strong pressure on the two miRNA–target regulatory pairs to be inherited together across ecotypes. Therefore, we can detect the presence or absence (co-occurrence) of the two regulatory pairs in the regulation fate profile. We defined a regulation fate profile to describe the occurrences, in the form of fate, of a certain miRNA–target regulation in the set of 1,135 ecotypes (see examples in Figures 1A–D): if miRNA*_i_*–target*_t_* and miRNA*_j_*–target*_t_* share the same regulation fate profiling, it indicates that miRNA*_i_* and miRNA*_j_* cooperatively regulate the target*_t_*.

Based on miRNA–target regulation at the population level, we initially identified miRNA–miRNA pairs that share a set of at least one target (*T*). Then, we estimated the extent to which miRNA*_i_* and miRNA*_j_* co-regulate target*_t_* ($t\in T\left(i,j\right)$), which was defined as a miRNA–miRNA–target co-regulation motif. We considered the ecotypes in which the two miRNAs and the target transcript are wild type or mutant type. In order to construct the regulation fate profile across ecotypes, we defined fate (*i*, *t*) as the fate between miRNA*_i_* and target*_t_* in an ecotype, and fate (*j*, *t*) between miRNA*_i_* and target*_t_*. For each shared target*_t_* in *T*(*i*, *j*) of miRNA*_i_* and miRNA*_j_*, we designed a co-regulation (*coreg*) score of this co-regulation motif (Formula 4, see examples in Figures 1A–D):


(4) $$\mathrm{coreg}\left(i,j,t\right)=\frac{\left|\mathrm{EcoK}\left(i,j,t\right)|+|\mathrm{EcoL}\left(i,j,t\right)|+|\mathrm{EcoG}\left(i,j,t\right)\right|}{\left|Eco\left(i,j,t\right)\right|}$$

where Eco (*i*, *j*, *t*) is the set of ecotypes containing wild- or mutant-type sequences of miRNA*_i_*, miRNA*_j_*, and target*_t_*. EcoK (*i*, *j*, *t*) is the set of ecotypes in which both regulatory pairs of miRNA*_i_*–target*_t_* and miRNA*_j_*–target*_t_* were kept, i.e., both fate (*i*, *t*) and fate (*j*, *t*) being k. EcoL (*i*, *j*, *t*) is the set of ecotypes in which both regulatory pairs were lost, i.e., both fate (*i*, *t*) and fate (*j*, *t*) being l. EcoG (*i*, *j*, *t*) is the set of ecotypes in which both regulatory pairs were gained, that is both fate (*i*, *t*) and fate (*j*, *t*) being g. Thus, *coreg* (*i*, *j*, *t*) is equal to the fraction of ecotypes in which both regulatory pairs present the same fates. From the definition, the values of co-regulation score are between 0 and 1. Clearly, *coreg* (*i*, *j*, *t*) = 0 indicates that the regulatory pairs of miRNA*_i_*–target*_t_* and miRNA*_j_*–target*_t_* have totally different regulation fate profile in all ecotypes; on the other hand, *coreg* (*i*, *j*, *t*) = 1 indicates that the two regulatory pairs show the same regulation fate profile. The larger the coreg value, the more possibility that miRNA*_i_* and miRNA*_j_* regulate the target*_t_* cooperatively. Note that in the case of the general fates of both regulatory pairs being KL (Figure 1C), we required that the two regulatory pairs were simultaneously lost in at least one ecotype, which means EcoL (*i*, *j*, *t*) was not empty, otherwise the co-regulation score equals to 0.

For each shared target*_t_* in *T*(*i*, *j*) of miRNA*_i_* and miRNA*_j_*, if the co-regulation score *coreg* (*i*, *j*, *t*) is smaller than a predefined threshold, the target*_t_* will be removed from *T*(*i*, *j*). Finally, if miRNA*_i_* and miRNA*_j_* co-regulate at least one shared target, the two miRNAs were considered cooperative; otherwise, this miRNA pair was deleted from the miRNA–miRNA crosstalk network.

### Definition of the crosstalk type for miRNA pairs

We called the combination of two miRNAs co-regulating a target as a miRNA–miRNA–target co-regulation motif. miRNA–target regulations were divided to dynamic (the general fate as G or KL) and static (the general fate as K) groups based on their general fates across ecotypes. Accordingly, we assigned a co-regulation type of “dd” (dynamic-dynamic), “ss” (static-static), or “sd” (static-dynamic) to each co-regulation motif. The co-regulation type of dd indicates that the two miRNAs dynamically co-regulate the target (both general fates as G or KL, Figures 1C,D), while ss indicates the two miRNAs statically co-regulate the target (both general fates as K, Figure 1A). The co-regulation type of sd indicates that one miRNA dynamically regulates a target, but the other keeps the regulation toward the same target in the reference genome and across ecotypes (one general fate as KL and the other as K, Figure 1B).

At the level of miRNA–miRNA crosstalk, we defined the crosstalk type for two miRNAs. If two miRNAs co-regulate multiple targets, we calculated the fractions of each co-regulation type among the motifs. If a particular co-regulation type belongs to more than 50% of the co-regulation motifs, it is assigned as the crosstalk type to the miRNA pair and written in capitals (see the example in Figure 1E). In other words, the crosstalk types of “DD,” “SS,” and “SD” for two cooperative miRNAs indicate more than half of the common targets are co-regulated with the co-regulation type of dd, ss, and sd, respectively.

To investigate the conservation preference of miRNA–miRNA pairs with different crosstalk types, randomization tests were performed to compare conservations among different crosstalk types of edges in the miRNA–miRNA crosstalk network. For each kind of crosstalk type, we randomly sampled the same number of edges as the observed network that are with this kind of crosstalk type. The randomization process was repeated 1,000 times. For each kind of crosstalk type, we compared the observed number of edges between two miRNAs within and between conservation groups to the expected number of edges in 1,000 randomized networks. A Z-score was calculated for each conservation group pair and each crosstalk type (Formula 5):


(5) $$Z-\mathrm{score}=\frac{X_{\mathrm{observed}}-X_{\mathrm{rand}}}{{SD}_{\mathrm{rand}}}$$

where $X_{\mathrm{observed}}$ is the number of edges between two miRNAs in an conservation group and with a crosstalk type, $X_{\mathrm{rand}}$ is the average number of edges between two miRNAs in the same conservation group and with the same crosstalk type in 1,000 random networks and ${SD}_{\mathrm{rand}}$ is the standard deviation of the numbers of edges from 1,000 random networks. *p*-values from the Z-scores were calculated with the function pnorm() in R software (R Core Team, 2014) and adjusted using Benjamini and Hochberg correction for multiple hypothesis testing.

### Evolutionary age of genes and miRNAs

The evolutionary age of *A. thaliana* genes was calculated as previously described (Defoort et al., 2018). *Arabidopsis thaliana* orthologous gene families were retrieved from PLAZA 5.0 dicots^3^ (Van Bel et al., 2018). They are derived from 98 fully sequenced species with a wide distribution over different evolutionary lineages. This resulted in 11 age groups: Cellular organisms, Eukaryota, Green plants/Viridiplantae, Land plants/Embryophyta, Vascular plants/Tracheophyta, Seed plants/Spermatophyta, Flowering plants/Magnoliopsida, Eudicots, Rosids, Brassicaceae, and *A. thaliana*. Each orthologous group was assigned an evolutionary age based on the oldest lineage of all the genes in the group, that is the earliest common ancestor of the orthologous group. For example, if an orthologous group contains one *A. thaliana* gene, two genes from species in the Brassicaceae lineage, and one gene from *Selaginella moellendorffii* in the vascular plant lineage, the age of this *A. thaliana* gene (or the whole orthologous group) is Vascular plants/Tracheophyta. The gene families, which have orthologs in other species other than *A. thaliana* were called “conserved genes”. The other genes only found in *A. thaliana* were called “non-conserved genes”. We obtained 26,117 (95% in all genes) conserved and 1,329 (5%) non-conserved genes.

To identify conserved miRNAs, the 428 *A. thaliana* miRNAs were searched against all the mature miRNAs of other species deposited in miRBase (release 22) using BLASTN. We chose the hits with *E*-value ≤0.01, ≥90% of the query and subject sequences covered, and ≤2 mismatches allowed. 200 (47%) *A. thaliana* miRNAs satisfying the above criteria were selected as “conserved miRNAs” while the other 228 (53%) miRNAs were specific to *A. thaliana* and called “non-conserved miRNAs.” Species in miRBase were also classified to the same eleven age groups, just like the species classification of protein-coding genes. Evolutionary ages of conserved miRNAs were estimated in a similar way as shown for protein-coding genes. The data shows that the group of Land plants is the oldest age of conserved miRNAs.

### Interaction homogeneity and age preference

Randomization tests were performed to compare age homogeneity among different general fates (K, KL and G) of edges in the population-level miRNA–target regulation network. 1,000 randomize networks were generated by randomly assigning each general fate to an edge in the original miRNA–target regulation network. The enrichment was calculated using Z-score and the *p*-value were corrected for multiple hypothesis testing. For the age homogeneity analysis in the miRNA–miRNA crosstalk network, we generated 1,000 randomized networks by permuting miRNA identifiers. The randomized networks have the same age and degree distributions as the observed network. Then, for each age group pair, we compared the observed number of interactions between the miRNAs to the expected number of interactions. A Z-score and a *p*-value were calculated based on this comparison. *p*-values were adjusted with Benjamini and Hochberg correction.

### miRNA expression profile data

We downloaded two miRNA expression profiling data produced through Illumina sequencing. The dataset GSE79414 (Xu et al., 2018) includes miRNA expression values in 27 different organ/tissue types, which cover the entire life cycle of *A. thaliana*. The dataset GSE66599 (Barciszewska-Pacak et al., 2015) includes miRNA expression values in a wide range of abiotic stress responses. Expression levels of miRNAs with their RPM values in all samples were downloaded. Expression values in multiple replicates for each sample were averaged. Expression values in each sample were log2 transformed. We combined the two datasets and removed miRNAs with expression values in <6 samples.

Pairwise Pearson’s expression correlation values between miRNAs were calculated and *p*-values were adjusted using Benjamini and Hochberg correction. Differences in the means of correlation coefficients among >2 miRNA–miRNA groups were analyzed using Analysis of Variance (ANOVA). TukeyHSD post-hoc method was used with the Benjamini and Hochberg correction.

### *Arabidopsis thaliana* genes and miRNAs in response to stress

We collected *A. thaliana* stress-responsive genes from five databases, PSGDb (Plant Stress Gene Database, 5,589 genes, http://bis.zju.edu.cn/PSGDb/), STIFDB2 (Stress Responsive Transcription Factor Database, 3,150 genes, http://caps.ncbs.res.in/stifdb2/; Naika et al., 2013), Plant Stress Gene Database (33 genes, http://ccbb.jnu.ac.in/stressgenes/frontpage.html), PRGdb 4.0 (Pathogen Receptor Genes, 2,819 genes, http://prgdb.org/prgdb4/; Calle Garcia et al., 2021), and DroughtDB (Drought Stress Gene Database, 101 genes, https://pgsb.helmholtz-muenchen.de/droughtdb/; Alter et al., 2015). The combined set contains 8,113 stress-related genes in *A. thaliana* involved in the plant tolerance to abiotic conditions (excessive or inadequate light, water, salt, temperature, ion and so on) and resistance to biotic conditions (insects and pathogen). *Arabidopsis thaliana* miRNAs related to their response to abiotic and biotic stress were obtained from PncStress^4^ (Wu et al., 2020). PncStress is a manually curated database of experimentally validated stress-responsive non-coding RNAs in plants. We obtained 162 *A. thaliana* miRNAs involved in plant tolerance to stresses including 28 abiotic stresses and four biotic stresses from 77 reports. Each miRNA is associated with one to 18 types of stress.

### Climatic data

Thousand one hundred thirty-one *A. thaliana* ecotypes among the 1,135 total ones have the latitude and longitude coordinates, which were used to query the WorldClim 2.1 database (released in January 2020, http://www.worldclim.org; Fick and Hijmans, 2017) for 19 climatic variables at a spatial resolution of 340 km^2^ (res = 10). The data was extracted using the raster package in R software. The climatic variables are based on yearly, quarterly, and monthly temperature and rainfall values. Principal component analysis of the climatic variables was conducted using the FactoMineR library in R (Supplementary Figure S1A). The first four principal components explained 83.9% of the variation. The first principal component was associated with temperature and the second principal component with precipitation. Pearson’s correlation coefficients between pairwise bioclimatic variables were calculated (Supplementary Figure S1B). In cases where variables were strongly correlated with one another, the variable with the most obvious link to the *A. thaliana* ecology was selected. Seven variables were used in the analyses, which are temperature seasonality, maximum temperature of warmest month, mean temperature of wettest quarter, mean temperature of coldest quarter, precipitation of wettest month, precipitation of driest month, and precipitation seasonality.

We used standard deviation to evaluate whether a group of ecotypes experienced similar climatic variables. For a climatic variable, the less the standard deviation from the average value, the more likely that the group of ecotypes share similar climatic values. To get a *p*-value, we randomly sampled the same number of ecotypes as the observed ecotype group, and repeated the permutation 1,000 times. The empirical *p*-value was calculated as the fraction of permutations that gave a smaller standard deviation value than that of our observed ecotype group. For multiple groups of ecotypes, the *p*-values were adjusted using Benjamini and Hochberg correction. A group of ecotypes share a similar climatic variable significantly under adjusted *p*-value <0.05.

### Network visualization and topological analysis

miRNA–miRNA crosstalk network was visualized in Cytoscape with yFiles organic layout (Shannon et al., 2003). Several topological features of the network were calculated using the “Analyze Network” tool (Assenov et al., 2008). Node degree measures the number of edges linked to a certain node. Node betweenness and edge betweenness are defined as the total number of nonredundant shortest paths going through a certain node and edge, respectively. Qualitatively, nodes with a high degree are considered as hubs, nodes with high betweenness are bottlenecks, and edges with high betweenness are bridges. To facilitate our calculations and discussion, we quantitatively defined hubs as the top 15% miRNAs with the highest degree values. Accordingly, bottlenecks were defined as the miRNAs that are in the top 15% in terms of node betweenness. Bridges are all edges in the top 15% of the edge betweenness values. To disentangle the topological and functional roles of hubs and bottlenecks, we divided all nodes in the network into four classes: hub-bottlenecks, hub-nonbottlenecks, nonhub-bottlenecks, and nonhub-nonbottlenecks (see the illustration in Supplementary Figure S2).

### Functional enrichment analysis of miRNAs

For a given miRNA, we first obtained a combined set of target transcripts co-regulated by this miRNA and its cooperative miRNAs. Then, we associated this miRNA with biological functions based on the functional enrichment analysis of the combined target set. Functional enrichment was conducted using clusterProfiler (v3.18.1; Yu et al., 2012; Wu et al., 2021) in R based on GO (Ashburner et al., 2000; Gene Ontology Consortium, 2021) biological process annotation. *Arabidopsis thaliana* whole-genome transcripts were taken as the reference set. clusterProfiler supports up-to-date gene annotation of thousands of species. Finally, we performed a REVIGO semantic relevance analysis to extract concentrated representative GO terms (Supek et al., 2011).

### Data availability

All of the public datasets and tools used in this study were listed in Supplementary Table S2.

## Results

### Static and dynamic miRNA–target pairs prefer to different age groups of miRNAs

To systematically delineate miRNA–target transcript regulation across multiple ecotypes, we generated a population-level miRNA–target regulation network using a multi-step method (see Materials and methods). It contains 9,036 miRNA–target pairs involving 371 miRNAs and 6,860 target transcripts encoded by 4,162 protein-coding genes from 1,135 ecotypes (Supplementary Table S3). For the reference genome of Col-0, there are 6,093 miRNA–target pairs involving 333 miRNAs and 4,572 target transcripts encoded by 2,791 protein-coding genes. Among the Col-0 miRNA-target pairs, 1,229 (20.2%) pairs contain reliable miRNA binding sites, which were validated by degradome-seq data (Supplementary Text S1; Supplementary Table S3; Supplementary Figure S3). The potential gain and loss of miRNA targets were obtained by comparing the miRNA–target pairs in the ecotypes with those in the reference genome (Formulas 1–3). Each miRNA–target pair was assigned a general fate as K for keeping present in the reference genome and all ecotypes, KL for presence in the reference genome and some ecotypes but absence in at least one ecotype, or G for absence in the reference genome but presence in at least one ecotype. The miRNA–target pair with the general fate of KL or G is called dynamic regulation, and the miRNA–target pair with the general fate of K is called static regulation. We obtained 3,813 miRNA–mRNA pairs with K, 2,280 with KL, and 2,943 with G.

**Figure 2:**
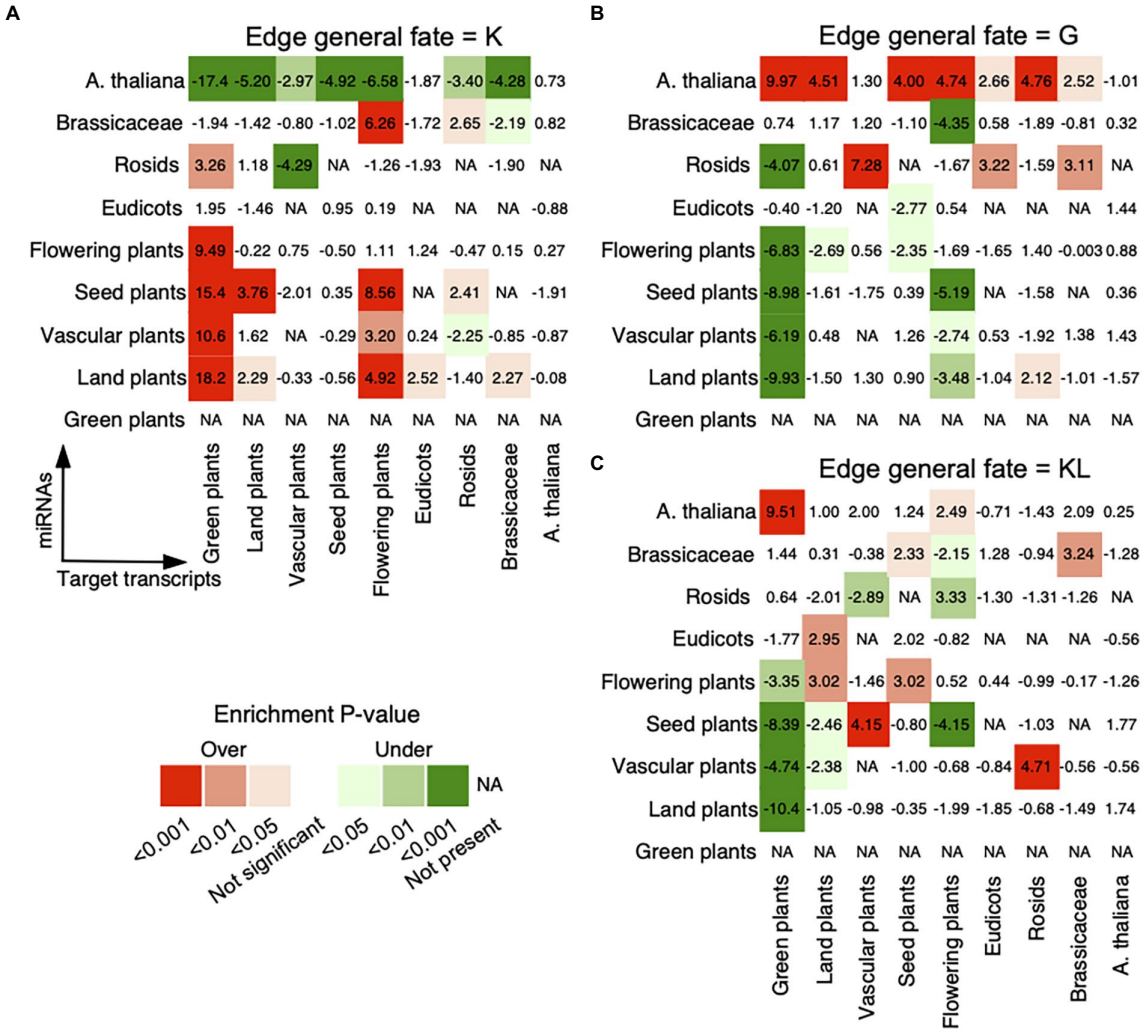
Age preference for miRNA–target regulations. The analysis was conducted for miRNA–target regulations with the general fates as **(A)** K, **(B)** G, and **(C)** KL. The observed number of regulatory pairs with the same fate within and between age groups in the real network was compared with the expected numbers in 1,000 randomized networks, which were generated by randomly assigning each general fate to an edge in the original network. The enrichment was calculated using Z-score (labeled within and between age groups) and the corrected *p*-value for multiple hypothesis testing (see Materials and methods). Overrepresentative results are shown in red and underrepresentative results are in green. The miRNAs are on the vertical axis and the target transcripts are on the horizontal axis. The oldest age of conserved miRNAs is Land plants.

To investigate the general age preference of the miRNA–target regulations, we analyzed whether regulations prefer miRNAs and transcripts of old or new evolutionary ages (see Materials and methods). For miRNA–target pairs with the general fate of K, we noted strong preferences of miRNAs from Flowering plants or older age groups for targeted transcripts from Brassicaceae or older age groups, of miRNAs from Rosids for transcripts from Green plants, and of miRNAs from Brassicaceae for transcripts from Flowering plants or Rosids (Figure 2A). For miRNA–target pairs with the general fate of G, miRNAs from *A. thaliana* were found to prefer to bind conserved transcripts, and miRNAs from Rosids preferred to bind transcripts from Vascular plants, Eudicots, or Brassicaceae (Figure 2B). For miRNA–target pairs with the general fate of KL, miRNAs from *A. thaliana* and Brassicaceae preferred to regulate conserved transcripts from Brassicaceae or older age groups. Besides that, we observed a strong preference of miRNAs from Eudicots or older age groups for conserved targets from Rosids or older age groups (Figure 2C). Hence, the static miRNA–target pairs, present in the reference genome and all ecotypes (with the general fate of K), are enriched between conserved miRNAs and conserved target transcripts. However, the dynamic regulatory pairs that are absent from the reference genome but gained in some ecotypes (with the general fate of G), show a different pattern from that of the static regulatory pairs and are overrepresented between non-conserved miRNAs and conserved targets. Interestingly, the miRNA–target pairs with the general fate of KL presented both patterns, in which both non-conserved and conserved miRNAs prefer to regulate conserved targets. Obviously, the general fates of miRNA–target pairs relate with the conservation of miRNAs, that is conserved (relating with the general fates of K and KL) and non-conserved (relating with the general fates of G and KL).

The correspondence of dynamic and static regulation fates of miRNA–target pairs to miRNAs with different conservation leads us to examine the difference in variant density between conserved and non-conserved miRNA classes. Since the miRNAs that contain any short indel (including short insertion or deletion variant) were deleted from the calculation of miRNA target prediction, only SNPs were considered in the miRNA sequences but both SNPs and short indels could be found in their upstream and downstream regions. Variant density of a region suggests the number of variants divided by the length of the region. We observed that the non-conserved miRNAs exhibited higher variant density than the conserved miRNAs (Mann–Whitney test, *p*-value = 3.9e-27; Supplementary Figure S4). The variant density is higher in the non-conserved miRNAs than in the upstream and downstream 5-kb regions (ANOVA, *p*-value <0.01), whereas the variant density is lower in the conserved miRNAs than in the upstream and downstream regions (*p*-value <2e-10; Supplementary Figure S4). A similar result has been reported in human (Han and Zheng, 2013) and rice (Liu et al., 2013). The miRNAs that keep the regulation toward target transcripts in the reference genome and the ecotypes (the general fate = K) are likely to be the conserved miRNA with a low SNP density. In contrast, the miRNAs that gain new targets in some ecotypes (the general fate = G) are enriched for the non-conserved ones with a high SNP density.

### Quality evaluation of miRNA–miRNA crosstalk

**Figure 3:**
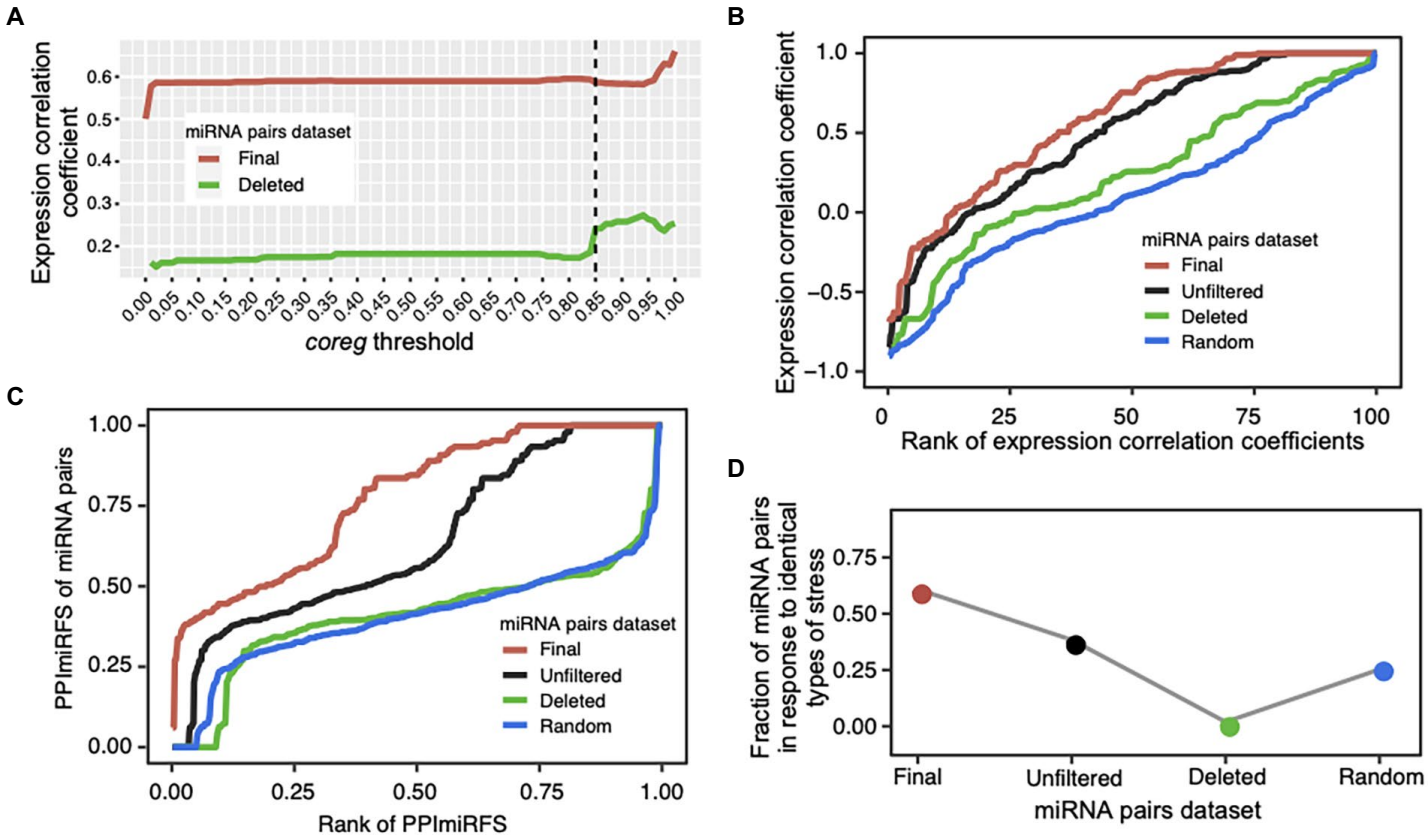
Validation of cooperative miRNA–miRNA pairs. **(A)** Average Pearson’s expression correlation coefficients of the miRNA pairs in the “final” (red line) and “deleted” (green line) datasets under a certain *coreg* threshold (0 ≤ *coreg* ≤ 1). The *coreg* threshold of 0.85 (dotted line) was used to filter the initial “unfiltered” miRNA pairs that share at least one target transcript. Under this threshold, the correlation coefficient of the miRNA pairs sharply increased in the “deleted” dataset. **(B)** Quality evaluation of the miRNA–miRNA crosstalk network using expression profiles. Correlation coefficient values were ranked increasingly for all miRNA pairs in the four datasets: “unfiltered”—the initial miRNA pairs dataset that share at least one target transcript (black line), “final”—the miRNA–miRNA crosstalk dataset after filtering the initial dataset using the *coreg* threshold of 0.85 (red line), “deleted”—the miRNA pairs that have no common target under the *coreg* threshold of 0.85 (green line), and “random”—the randomized miRNA pairs with the same topology as the “final” network, and generated by shuffling the node labels while keeping the edges constant (blue line). The “final” dataset shows the significantly highest expression correlation coefficient. **(C)** Evaluation of miRNA pairs using PPImiRFS. PPImiRFS scores were ranked increasingly in each dataset. PPImiRFS was used to infer the functional similarity scores of miRNA pairs based on a protein–protein interaction network with semantic similarity weights generated using GO terms and graph theoretical properties (Meng et al., 2015). The “final” dataset corresponds to the highest PPImiRFS scores. **(D)** Fraction of the miRNA pairs in response to the identical type of stress. The “final” dataset obtained the highest fraction of miRNA pairs responsive to the identical type of stress, and the “deleted” pairs showed the lowest value.

**Figure 4:**
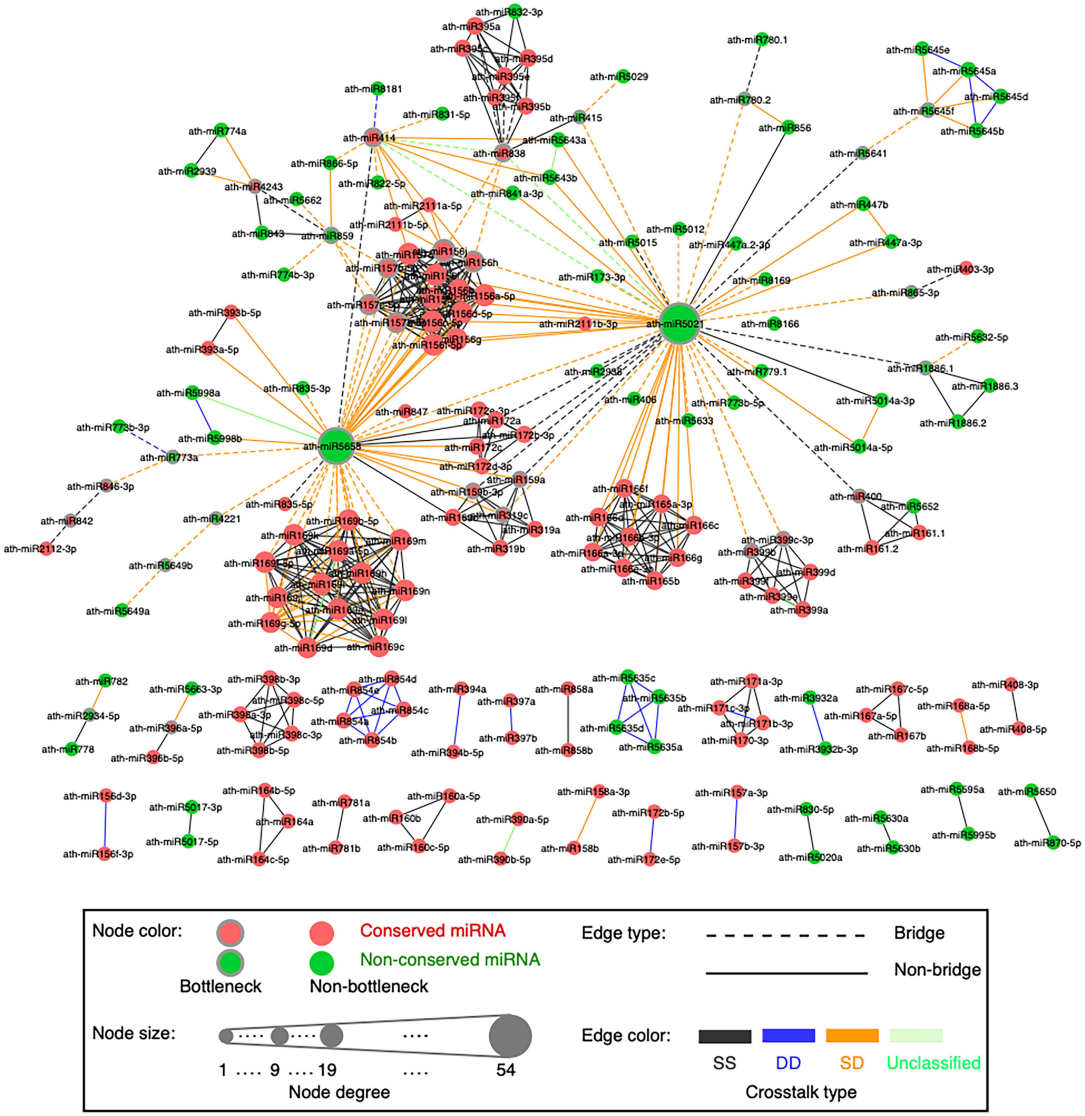
Global visualization of the miRNA–miRNA crosstalk network. A circle node represents miRNA. Node size is proportional to the degree of miRNAs. Bottleneck nodes (the top 15% miRNAs with the highest node betweenness values) are in the gray border. Red nodes indicate conserved miRNAs, while green nodes are non-conserved miRNAs. An edge represents crosstalk between two miRNAs. Edges in dotted lines indicate bridges (the top 15% edges with the highest edge betweenness values). Edges are colored according to the crosstalk types, which are SS type in black, DD type in blue, SD type in orange, and unclassified type in light green.

We designed a *coreg* score to estimate the possibility that two miRNAs co-regulate a common target based on their regulation fate profiles (see Materials and methods; Figures 1A–D). We defined a wide range of *coreg* thresholds from 0 to 1 in order to identify miRNA–miRNA crosstalk across ecotypes. If two miRNAs regulate a target with the *coreg* score smaller than the threshold, the target will be removed from the common target set. Under each threshold, the initial “unfiltered” miRNA–miRNA pairs that share at least one target were divided to two miRNA pair datasets, namely the “deleted” miRNA pairs where all common targets were removed and the “final” miRNA pairs where at least one target was kept. Based on the expression compendium combined from the miRNA expression values of diverse organ/tissue types and abiotic stress responses, average Pearson’s correlation coefficients between two miRNAs in the two datasets were calculated (see Materials and methods; Figure 3A). The average expression correlation coefficients between miRNA pairs in the “final” and “deleted” datasets generally increased, suggesting that the larger the *coreg* values are, the higher expression correlations the miRNA pairs have. We observed that the expression correlation coefficients in the “deleted” dataset increased sharply from 0.18 to 0.24 when the *coreg* threshold was 0.85, but the increase slowed down after that. Thus, the *coreg* threshold of 0.85 was set to filter the initial miRNA–miRNA pair dataset that share at least one target. The constructed miRNA–miRNA crosstalk network contains 200 miRNAs and 506 cooperative interactions which co-regulate 612 transcripts (Figure 4, see detailed node and edge information in Supplementary Table S4). The miRNA–miRNA pairs co-regulate 10.8 transcripts on average with the maximum number of 48 (Supplementary Figure S5A). Specifically, for 382 (62.4% in 612) transcripts, each is co-regulated by only one miRNA–miRNA pair, while each of the remaining 230 transcripts is co-regulated by two to 105 miRNA–miRNA pairs (Supplementary Figure S5B).

Our network presents scale-free characteristics (Supplementary Figure S6A), indicating that most miRNAs are poorly connected and a few miRNAs are connected with a relatively large number of miRNA partners. The miRNA–miRNA synergistic networks in human (Hua et al., 2014; Shao et al., 2019) and plants (Xu et al., 2014; Banerjee and Mal, 2020) also show the scale-free structure. We disentangled the topological and functional roles of two complementary topological properties, namely hubs and bottlenecks (see Materials and methods; Supplementary Text S2). Bottlenecks tend to be more important mediators for network communication than nonbottlenecks in the miRNA–miRNA crosstalk network (Supplementary Figure S6B). miRNAs in the network might regulate the transcripts that participate in plant developmental process, reproduction, and signaling pathways (Supplementary Figure S6C).

To evaluate the performance of our method, we test the identified cooperative miRNA–miRNA pairs in terms of (1) expression correlations, (2) PPImiRFS scores, and (3) tendency to respond to the same type of stress. It has been revealed that most of synergistic miRNA pairs tend to be co-expressed, which may help make a rapid response to external disturbances (Zhang et al., 2019). We compared the expression correlation coefficients of the “final” miRNA pairs with those of the “unfiltered,” “deleted” and randomized (“random”) datasets. “Unfiltered” dataset contains the initial miRNA pairs (818 pairs among 270 miRNAs) that co-regulate at least one target before calculating the *coreg* score. As shown in Figure 3B, the mean correlation coefficients of the “final” dataset (mean = 0.59 ± 0.02 also shown in Figure 3A, 0.02 is the standard error) is significantly higher than those of the other three datasets (ANOVA, mean = 0.5 ± 0.02 for the “unfiltered” dataset and *p*-value = 5e-8, mean = 0.24 ± 0.04 for the “deleted” dataset and *p*-value = 8e-13, and mean = 0.1 ± 0.02 for the “random” dataset and *p*-value <2e-16). Expression correlations for the “final” miRNA pairs are listed in Supplementary Table S4. Furthermore, we classified miRNA–miRNA pairs into three groups according to the conservation of miRNAs, and assessed the density of expression correlation values in different groups. Except for the non-conserved and conserved miRNA–miRNA group, the “final” pairs showed a stronger expression correlation than the “deleted” and “random” ones (ANOVA, all *p*-values <0.05; Supplementary Figure S7). Previous studies have shown that conserved and non-conserved miRNAs have different expression patterns in *A. thaliana*. Conserved miRNAs tend to be constitutively activated while expression of non-conserved ones is more organ-specific (Yang et al., 2011; Xu et al., 2018). In addition, the expression levels of conserved miRNAs are much higher than non-conserved ones across organs/tissues types (Xu et al., 2018). These differences in expression patterns between conserved and non-conserved miRNAs may account for the significant lower expression correlation of the “final” miRNA pairs in the conserved and non-conserved group (Mean = 0.1 ± 0.03) than those in the other two conservation groups (ANOVA, mean = 0.66 ± 0.02 for conserved and conserved pairs and *p*-value <2e-16, mean = 0.58 ± 0.08 for non-conserved and non-conserved pairs and *p*-value = 4e-7; Supplementary Figure S7).

The functional similarity scores of miRNAs in *A. thaliana* have been inferred from the functional similarity of their target sets using the PPImiRFS method (Meng et al., 2015). A protein–protein interaction network with semantic similarity weights of edges generated using GO terms was constructed and the functional similarity scores were calculated using graph theoretical properties (Meng et al., 2015). To verify our results, PPImiRFS scores were compared in the above four miRNA–miRNA datasets (Figure 3C, PPImiRFS scores for the “final” miRNA pairs are in Supplementary Table S4). The average PPImiRFS score of the “final” dataset (mean = 0.78 ± 0.01) is significantly higher than those of the other three datasets (ANOVA, all *p*-values = 6.6e-11, mean = 0.64 ± 0.01 for the “unfiltered” dataset, mean = 0.41 ± 0.01 for the “deleted” dataset, and mean = 0.41 ± 0.01 for the “random” dataset). There is no difference of PPImiRFS for the miRNA–miRNA pairs between the “deleted” and “random” datasets (Figure 3C). Thus, PPImiRFS clearly verify the utility of our method for producing cooperative miRNA pairs.

Meng et al. (2015) reported that the miRNA pairs responding to the same type of stress have higher functional similarity than the miRNA pairs responding to different types of stresses. We gathered a set of 162 miRNAs of *A. thaliana*, which were experimentally verified to be associated with stress responses (see Materials and methods; Supplementary Table S2). Firstly, we investigated whether our miRNA–miRNA crosstalk network is enriched with stress-responsive miRNAs. Compared with the 162 stress-responsive miRNAs out of the 428 *A. thaliana* miRNAs registered in miRBase, 108 stress-responsive miRNAs were identified from our miRNA pairs among 200 miRNAs. Thus, the miRNA–miRNA crosstalk network is significantly enriched with miRNAs in response to stress (Fisher’s exact test, *p*-value = 8e-11). Notably, 91% (147 out of 162 total ones) of stress-responsive miRNAs are conserved, whereas only 20% of the miRNAs that have not been reported to respond to stresses, are conserved. Thus, stress-responsive miRNAs tend to be conserved (Fisher’s exact test, *p*-value = 2e-50). We further observed that 308 (60.9% of 506 total pairs) of our identified miRNA–miRNA pairs respond to the same types of stresses, which is significantly greater than that in the “unfiltered,” “deleted,” and “random” datasets (Figure 3D, stresses shared by the miRNA pairs are also listed in Supplementary Table S4). Therefore, the miRNA–miRNA crosstalk network is enriched with miRNA pairs responding to identical stress, indicating they are involved in similar functions.

In conclusion, our identified cooperative miRNA pairs tend to be highly co-expressed, respond to identical types of stresses, and associated with high PPImiRFS scores. The miRNA–miRNA crosstalk network is reliable and enriched for genuine positive.

### miRNA–miRNA crosstalk preferentially occurs between miRNAs of similar age

**Figure 5:**
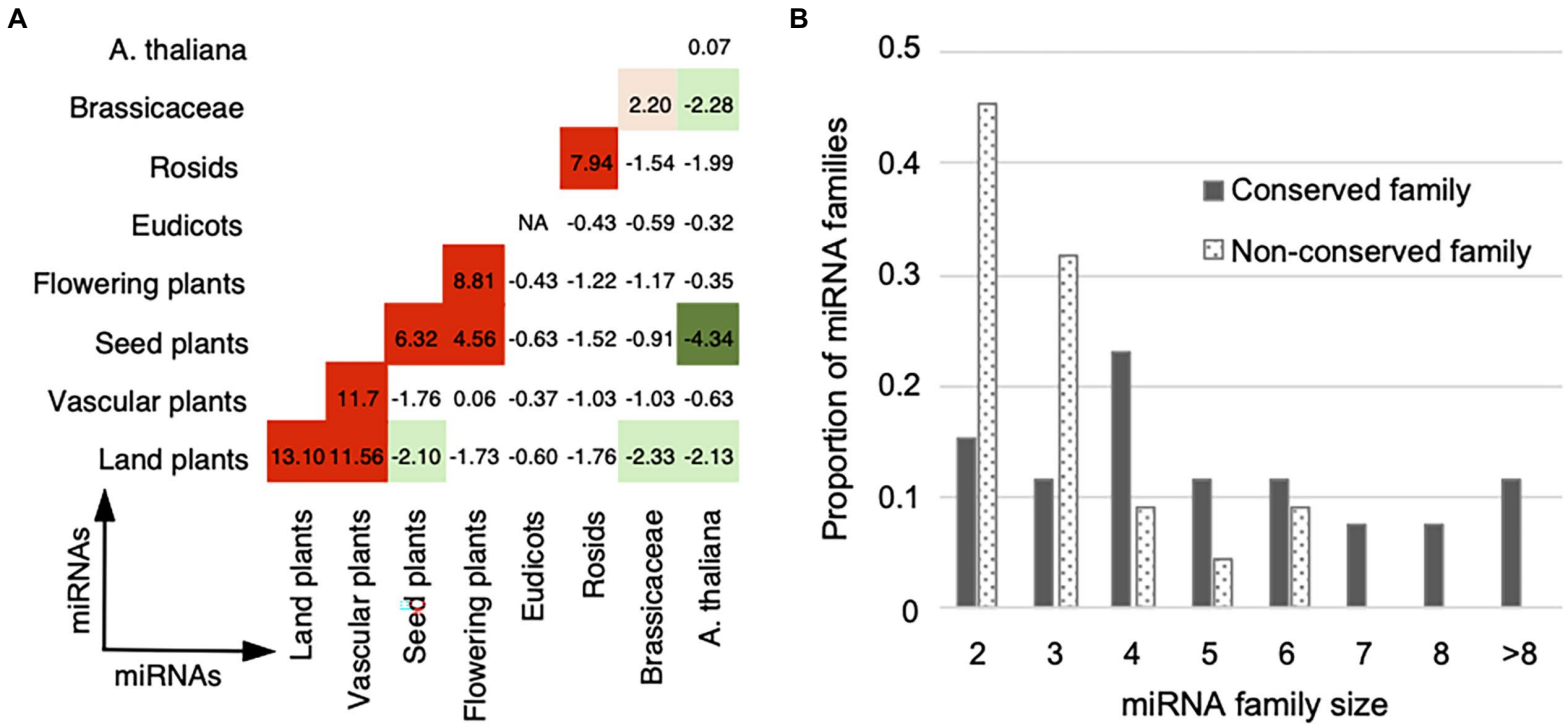
Cooperation age preference for miRNA pairs. **(A)** Cooperation age preference for miRNA pairs. The observed number of cooperative miRNA pairs within and between age groups in the real network was compared with the expected numbers in the 1,000 randomized networks with the same degree and age distributions (see Materials and methods). The enrichment was calculated using Z-score (labeled within and between age groups) and the corrected *p*-value for multiple hypothesis testing. Overrepresentative results are shown in red and underrepresentative results are in green (see the same legend in Figure 2). **(B)** Distribution of the family size for the conserved (gray bar) and non-conserved (dotted bar) miRNA families. The conserved miRNA families are of larger size than the non-conserved ones.

To assess the interaction preference of miRNA pairs in different conservation groups, we analyzed the conservation enrichment in the “final,” “deleted,” and “unfiltered” datasets, and then compared with the “random” dataset (Supplementary Table S5). The “final” and “unfiltered” datasets attract more conserved and conserved miRNA pairs than expected by random (*Z*-test, *p*-value = 3.9e-7 for the “final” and *p*-value = 4.9e-5 for the “unfiltered”), and are underrepresented with miRNA pairs with different conservations, that is conserved and non-conserved pairs (*Z*-test, *p*-value = 3.1e-22 for the “final” and *p*-value = 1.4e-37 for the “unfiltered”). Furthermore, we analyzed whether cooperative miRNA pairs in the “final” dataset prefer to interact within or between age groups. Figure 5A shows high Z-scores are found on or near the main diagonal of the age group matrix, demonstrating a crosstalk preference toward the own age group or to the next age groups.

MiRNAs belonging to the same family show more similar functions than the miRNAs of different families, which have been supported by the miRNA–miRNA crosstalk networks in different species (Xu et al., 2011; Sun et al., 2013; Chen et al., 2014; Meng et al., 2015; Shao et al., 2019). We inferred the relationship between the cooperative miRNA pairs and the miRNA families. Our miRNA–miRNA crosstalk network contains 284 intra-family miRNA pairs and 222 inter-family pairs. Compared with the numbers of all possible intra- and inter-family pairs derived from the miRNAs in miRBase, our network is enriched with homologous miRNA pairs, that is miRNA–miRNA crosstalk within miRNA families (Fisher’s exact test, *p*-value = 1.9e-316). According to the conservation of miRNA members, 48 multiple-member families were divided into 26 conserved ones (>50% members are conserved) and 22 non-conserved ones (>50% members are non-conserved). 88% (23 out of 26) of the conserved families contain homologous miRNA pairs that are in our miRNA–miRNA crosstalk network, whereas the proportion of homologous miRNA pairs in the non-conserved families decreased to 59% (13 out of 22). In addition, the conserved families are of larger size than the non-conserved ones (Figure 5B; Mann–Whitney test, *p*-value = 0.002). Interestingly, the miRNA families that contain the largest number of cooperative homologous miRNA pairs are conserved families. Five conserved families (ath-miR156/157, ath-miR169, ath-miR165/166, ath-miR159/319, ath-miR395, and ath-miR172) contain 11–93 cooperative homologous miRNA pairs, comprising 40% to 100% of all possible intra-family member pairs (Supplementary Table S6). These results suggest that conserved and non-conserved miRNA families are somewhat different: the conserved miRNA families are of larger size and therefore contribute more to the crosstalk among miRNAs.

### Static and dynamic miRNA–target regulations contribute to the cooperative miRNA pairs acting various biological characteristics

To understand how two miRNAs cooperatively interact to regulate a group of transcripts across ecotypes, we defined the crosstalk type as DD, SS, or SD for each miRNA pair. The example showing the definition of the DD crosstalk type between ath-miR172b-5p and ath-miR172e-5p was displayed in Figure 1E. For ath-miR400 and ath-miR161.2 co-regulating 13 transcripts, 12 transcripts are co-regulated with an co-regulation type of ss across ecotypes and thus SS is considered as the crosstalk type between the two miRNAs. As a result, there are 336 SS (gray edge in Figure 4), 31 DD (blue edge), 131 SD (orange edge) and eight unclassified (green edge) cooperative miRNA pairs in the network (Supplementary Table S4).

**Figure 6:**
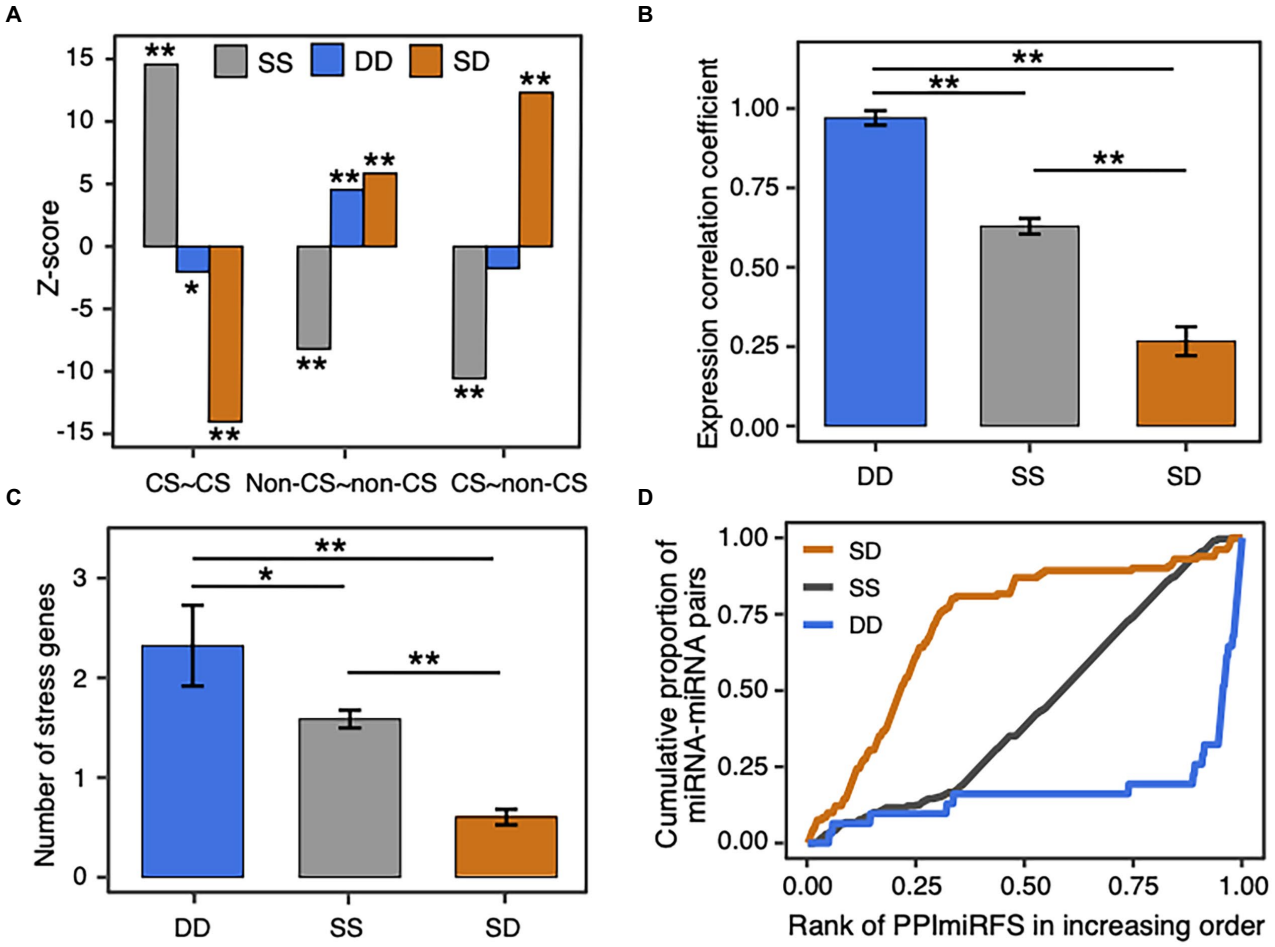
Biological significance of SS, DD, and SD miRNA–miRNA crosstalk. **(A)** Z-score distribution of the SS, DD, and SD miRNA pairs in different conservation groups. miRNAs are classified into conserved (CS) and non-conserved (non-CS) groups. The observed number of miRNA pairs with the same crosstalk type between conservation groups in the real network was compared with the expected number in the 1,000 randomized networks, which were generated by randomly assigning each crosstalk type to an edge in the original network (see Materials and methods). The enrichment was calculated using Z-score and the corrected *p*-value for multiple hypothesis testing. The symbol ^*^ denotes *p*-value < 0.01 and ^**^ denotes *p*-value < 0.001. **(B)** Expression correlation of miRNA pairs with different crosstalk types. Data are represented as the average Pearson’s correlation coefficient ± standard error. The symbol ^*^ denotes *p*-value < 0.01, and ^**^ denotes *p*-value < 0.001 using Analysis of Variance (ANOVA) with the Tukey HSD post-hoc method and the Benjamini and Hochberg correction. **(C)** Number of stress genes co-regulated by the SS, DD, and SD miRNA pairs. **(D)** Distribution of the cooperative miRNA pairs in different ranks of PPImiRFS scores. PPImiRFS scores were sorted increasingly.

Since the static miRNA–target regulations are enriched with the conserved miRNAs and the dynamic miRNA–target regulations favor the non-conserved miRNAs (Figure 2), we investigated the relationship between miRNA–miRNA crosstalk types and miRNA conservations. As a result, SS cooperative miRNA pairs prefer the conserved and conserved pairs (*p*-value = 2.5e-47), DD miRNA pairs prefer the non-conserved and non-conserved pairs (*p*-value = 4.9e-6), and SD miRNA pairs are enriched in the conserved and non-conserved pairs (*p*-value = 1.4e-34), as well as the non-conserved and non-conserved pairs (*p*-value = 5e-9; Supplementary Figure S8; Figure 6A). Thus, the conservation pattern of the miRNA pairs with different crosstalk types relates well with the conservation preference of miRNA–target regulations. Considering that conserved and non-conserved miRNA pairs have lower expression correlation than the other pairs in the miRNA–miRNA crosstalk network (red lines in Supplementary Figure S7), we compared expression correlation between the miRNA pairs with different crosstalk types. Expectedly, the SD miRNA pairs have the lowest expression correlation (mean = 0.27 ± 0.05, ANOVA, *p*-value <1.2e-10; Figure 6B), which is in agreement with their preference for non-conserved and conserved miRNA pairs. Interestingly, the DD miRNA pairs are better co-expressed than the SS miRNA pairs (mean = 0.97 ± 0.02 for DD pairs, mean = 0.63 ± 0.02 for SS pairs, *p*-value = 1.2e-4). In addition, global view of the miRNA–miRNA crosstalk network and Fisher’s exact tests showed that DD (*p*-value = 3e-6, odds ratio = 12.4) and SS (*p*-value = 6e-14, odds ratio = 4.3) miRNA pairs are more likely to be homologous miRNA pairs, whereas the SD miRNA pairs tend to be in different families (*p*-value = 8e-26, odds ratio = 0.09). Furthermore, 84% (281 out of 336 SS ones) SS miRNA pairs respond to the identical type of stress and the number is significantly larger than expected by random (empirical *p*-value = 0; see Materials and methods). By contrast, only 19% DD pairs and 14% SD pairs respond to the identical type of stress (Supplementary Table S7 and see the detailed information in Supplementary Table S4). Therefore, the miRNA–miRNA pairs responding to the identical type of stress favor SS interactions, which may be attributed to the enrichment of stress-responsive miRNAs in the conserved ones. In general, the cooperative miRNA interactions with different crosstalk types present various biological characteristics in terms of miRNA conservation, expression, homology, and stress response.

An edge with a high edge betweenness centrality indicates that it acts as a bridge-like connector between two subgraph clusters. Deletion of the bridge may affect the communication between many pairs of nodes through the shortest paths divided by the two subgraph clusters (Girvan and Newman, 2002). By taking the whole network as the background, we found the SD miRNA pairs (orange edges in Figure 4) are enriched for bridge edges (dotted edges in Figure 4; Fisher’s exact test, *p*-value = 8.8e-18, odds ratio = 9.5). The majority of SD miRNA pairs involve the three hub-bottlenecks, ath-miR5021, ath-miR5658, and ath-miR414, that are with the highest degree and the highest node betweenness. Another example is two SD miRNA pairs, one between ath-miR838 and ath-miR156j, and the other between ath-miR838 and ath-miR156h, are bridges linking two inter-connected subgraph clusters (Figure 4; Supplementary Figure S9). Bridge edges preferentially link two bottleneck miRNAs (Fisher’s exact test, *p*-value = 1e-16), emphasizing that the bridges take an important topological role in connecting different subgraphs together. In addition, edge betweenness values of the cooperative miRNA pairs are negatively correlated with their expression correlations (Pearson’s correlation, *p*-value = 2e-11), which is consistent with the observation that the SD miRNA pairs have a much lower expression correlation than the other types of cooperative miRNA pairs in the network (Figure 6B). These suggest that the SD miRNA pairs may act as the bridges between two different subgraph clusters to enhance their communications.

For the DD miRNA–miRNA crosstalk, the two miRNAs’ regulations toward their common targets were gained or dropped together in the same ecotypes. We investigated whether the DD miRNA pairs target more stress-responsive genes than the miRNA pairs with the other crosstalk types. To this end, we combined five databases of stress-responsive genes in plants and obtained 8,113 abiotic and biotic stress-responsive genes in *A. thaliana* (see Materials and methods). A total of 129 stress-responsive genes are targeted by cooperative miRNA pairs in our network (Supplementary Table S4). The DD miRNA pairs co-regulate the most stress genes compared with the SS (ANOVA, *p*-value = 0.02) and SD (*p*-value = 9e-8) miRNA pairs. SS-type miRNA pairs co-regulate more stress genes than SD miRNA pairs (*p*-value = 3e-9; Figure 6C). The ath-miR854 family members cooperatively regulate five stress genes, including *AT1G80440* and *AT4G37180* encoding transcription factors involved in the abiotic stress (drought, cold, and salt) responses, *AT1G15530* and *AT4G23210* as disease resistance genes involved in defense response to bacterium, and *AT3G10630* encoding DP-glycosyltransferase superfamily protein. The miRNA expression profiling data produced by Barciszewska-Pacak et al. (2015) (GSE66599) showed that the ath-miR854 family members were significantly down-regulated under drought stress and slightly up-regulated under high-salinity stress, supporting our result that the ath-miR854 members cooperate with each other to regulate stress-responsive genes (Supplementary Table S8). Another example includes ath-miR172b-5p and ath-miR172e-5p with the DD crosstalk type. The two miRNAs co-regulate four stress-responsive genes, including *AT2G16500* (encoding arginine decarboxylase 1, ADC1) involved in response to abiotic stresses (cold, drought, ion, and salt), *AT5G48410* (glutamate receptor 1.3, GLR1.3) involved in response to ion and light stimuli, *AT3G57330* (auto-inhibited Ca2+-ATPase 11, ACA11) participated in defense response to bacterium, and *AT2G22950* (auto-inhibited Ca2+-ATPase 7, ACA7). The miRNA expression data by Barciszewska-Pacak et al. (2015) showed that both ath-miR172b-5p and ath-miR172e-5p were significantly up-regulated under drought and high-salinity stresses, and also slightly down-regulated under copper deficiency, consisting with our finding from the miRNA–miRNA crosstalk network (Supplementary Table S8).

### Case studies for the dynamic state of miRNA–target regulations in specific ecotypes

Since both mutant-type miRNAs and mutant-type transcripts for each ecotype were considered in predicting miRNA–target regulations, SNPs within the mature miRNAs or the miRNA binding sites on the targets could cause gain or loss of the regulatory pairs in specific ecotypes (Gong et al., 2012). In a miRNA–miRNA–target co-regulation motif, SNPs within the miRNA binding site on the target could affect both regulatory pairs, while SNPs within one miRNA sequence may impact the regulation by itself. For the dd co-regulation motifs, in which both miRNA–target regulatory pairs are reprogrammed in specific ecotypes, are the dynamic state of both regulatory pairs likely to be associated with the SNPs within the miRNA binding site on the target? For the sd co-regulation motifs, in which only one miRNA–target regulation is reprogrammed, is the dynamic state of this regulatory pair likely to result from the SNPs within the miRNA that dynamically regulates the target? Indeed, 78% of the sd co-regulation motifs in our network are disrupted because of the SNPs in the mature miRNA sequences, 15% are attributed to the SNPs in miRNA binding sites, and the left 7% are associated with the SNPs in both kinds of regions. However, among the dd co-regulation motifs, 95% are dynamically gained or lost because of the SNPs in the miRNA binding sites on targets and only 3% are caused by the SNPs in the mature miRNAs (See examples in Supplementary Text S3). Therefore, the dynamic state of miRNA–target regulations in the sd and dd co-regulation motifs are likely to be associated with the SNPs within the miRNA sequence and the miRNA binding site, separately. miRNA regulation could be reprogrammed in different genomes. We then investigated the ecotypes, in which the multiple dynamic regulations toward each transcript are gained or lost, and discussed whether these ecotypes are exposed to similar or diverse climatic conditions. Three case studies were also presented as follows.

**Figure 7:**
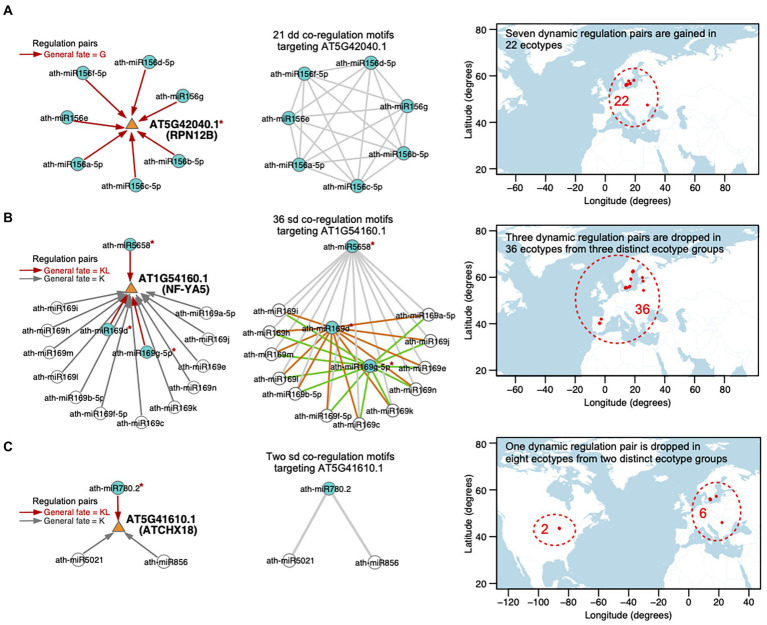
Case studies for the dynamic miRNA regulations toward the targets, that are gained or dropped in specific ecotypes. Three transcripts are shown as examples, which are **(A)** AT5G42040.1 (RPN12B), **(B)** AT1G54160 (NF-YA5), and **(C)** AT5G41610.1 (ATCHX18). **(Left panel)** miRNA–target regulations. The targeted transcript is depicted with an orange triangle and the miRNA is depicted with a circle. Directed edges represent miRNA–target regulations, including dynamic regulations (the general fate = G or KL, red edge) and static regulations (the general fate = K, gray edge). A blue circle indicates that the corresponding miRNA dynamically regulates the target, while a white circle represents a static regulation toward the target. The red symbol * beside a miRNA or a target denotes the reprogramming of the regulation attributed by the SNP(s) in the sequence. **(Middle panel)** Cooperative miRNA pairs co-regulating a transcript, defined as miRNA–miRNA–target co-regulation motifs. A circle node represents miRNA and an edge represents a crosstalk between two miRNAs. There are 21 dd-type, 36 sd-type, and 2 sd-type co-regulation motifs targeting **(A)** AT5G42040.1, **(B)** AT1G54160.1, and **(C)** AT5G41610.1, respectively. dd-type co-regulation motifs toward AT5G42040.1 are newly gained in the same 22 ecotypes, which is related to an SNP in the target **(A)**. In contrast, sd-type co-regulation motifs are disrupted in non-overlap ecotype groups because of the SNPs in **(B)** different miRNAs sequences or **(C)** different SNPs in the same miRNA sequence. **(Right Panel)** Map showing the locations (longitude on x-axis and latitude on y-axis) from which the ecotypes employed in each case study originate. The region harboring the ecotypes (red dots) is included in a red dashed circle with the number of ecotypes labeled. For each ecotype group, the standard deviation values and adjusted empirical *p*-values of seven bioclimatic variables are listed in Supplementary Table S9.

There are 230 transcripts co-regulated by multiple miRNA pairs. Among them, 32 transcripts are involved in multiple dd miRNA–miRNA–target co-regulation motifs. The dynamic regulations toward the 32 transcripts are gained or dropped because of the SNPs within the miRNA binding sites on targets. Moreover, the multiple dynamic regulations toward each transcript are gained or dropped in the same ecotype groups. Taking AT5G42040.1 (RPN12B, regulatory particle non-ATPase 12B) as an example, it is regulated by seven members of the ath-miR156 family, constituting 21 dd co-regulation motifs (in the left and middle panels of Figure 7A; Supplementary Table S9). All of the seven miRNAs gain the target transcript in the same 22 ecotypes because of the SNP (T-to-A) in the 3′UTR located within the miRNA binding site on AT5G42040.1. Among the 22 ecotypes, 21 ecotypes are distributed in Sweden and the remaining one is in Romania (in the right panel of Figure 7A). Interestingly, the 22 ecotypes are exposed to a very similar temperature and precipitation conditions, evaluated with all of the seven climatic variables (see Materials and methods; Supplementary Table S9).

In addition, there are 65 transcripts involved in multiple sd co-regulation motifs. 27 (42%) transcripts are dynamically regulated by at least two miRNAs with fate as KL and the dynamic regulations toward these targets are related to SNPs in the miRNA sequences. We observed that the SNPs in the multiple miRNAs that dynamically regulate the same target lead to a drop of the regulations in different ecotype groups, which may have overlaps (20 out of 27 targets) or have no overlap (7 out of 27 targets). For instance, AT1G54160.1 (NF-YA5, a CCAAT-binding transcription factor up-regulated by ABA and drought) is regulated by the hub-bottleneck ath-miR5658 and 14 members of the ath-miR169 family, constituting 36 sd co-regulation motifs (in the middle panel of Figure 7B; Supplementary Table S9). Only ath-miR5658, ath-miR169d, and ath-miR169g-5p dynamically regulate AT1G54160.1 (the general fate = KL), while the other miRNAs regulate the target with the general fate as K (in the left panel of Figure 7B). The regulation between ath-miR5658 and AT1G54160.1 is lost in 32 ecotypes due to the SNP at position 17 (T-to-A) in ath-miR5658, which leads to the disruption of 12 corresponding sd-type co-regulation motifs (gray edges in the middle panel of Figure 7B) in these 32 ecotypes. The regulation between ath-miR169d and AT1G54160.1 is lost in another two ecotypes due to the SNP at position 9 (G-to-T) in ath-miR169d, which contributes to the disruption of another 12 sd-type co-regulation motifs (orange edges in the middle panel of Figure 7B). The regulation between ath-miR169g-5p and AT1G54160.1 is lost in the other two ecotypes due to the SNP at position 13 (G-to-A) in ath-miR169g-5p, which results in a collapse in the other 12 sd-type co-regulation motifs (green edges in the middle panel of Figure 7B). Furthermore, the three ecotype groups have no overlap. The 36 ecotypes are distributed in Spain, Sweden, Republic of Lithuania, and Republic of Estonia (in the right panel of Figure 7B) but are exposed to very similar temperature (maximum temperature of warmest month, mean temperature of wettest quarter) and precipitation (precipitation of wettest month, precipitation of driest month, and precipitation seasonality) conditions (Supplementary Table S9).

Different SNPs in the same miRNA sequence or in the same miRNA binding site probably have distinct effects on the miRNA–target regulation across ecotypes. 34 (58% of 65 ones) targets were involved in multiple sd co-regulation motifs and dynamically regulated by only one miRNA. Different SNPs within the miRNA (associated with eight targets) or the binding site on the target (associated with four targets) are likely to lead to the drop of the dynamic regulation in distinct ecotype groups. AT5G41610.1 (ATCHX18, member of Putative Na+/H+ antiporter family) is co-regulated by two miRNA–miRNA pairs, ath-miR780.2 (the general fate = KL) and the hub-bottleneck ath-miR5021 (the general fate = K), as well as ath-miR780.2 and ath-miR856 (the general fate = K; in the middle panel of Figure 7C; Supplementary Table S9). The regulation between ath-miR780.2 and AT5G41610.1 is lost in eight ecotypes, which is divided to two distinct groups. The regulation between ath-miR780.2 and AT5G41610.1 is dropped in two ecotypes distributed in United States, which is related to two SNPs at positions 1 (T-to-A) and 2 (T-to-G) in the ath-miR780.2 sequence. It is also dropped in the other six ecotypes distributed in Sweden and Romania, which is related to another SNP at the position 6 (C-to-A) in ath-miR780.2. Interestingly, the eight ecotypes are distributed at similar latitudes (43–57 degrees N; in the right panel of Figure 7C), and are exposed to environments sharing similar precipitation of driest month and precipitation seasonality (Supplementary Table S9).

## Discussion

### The regulations toward a target may be reprogrammed in ecotypes exposed to similar climatic conditions

It is worthy to note that the general fate of a certain miRNA–target regulation across ecotypes compared with its occurrence in the reference genome can be classified as dynamic (the general fate as G or KL) or static (the general fate as K) class. We observed the dynamic fate in dd miRNA–miRNA–target co-regulation motifs is likely to be associated with SNPs in the miRNA binding sites on targets, whereas the dynamic fate in sd co-regulation motifs is mainly attributed to SNPs in the miRNA sequences. Different SNPs in the same miRNA sequence or miRNA binding site region may lead to the dynamic miRNA–target regulation being lost or newly gained in different ecotype groups, which have overlaps or no overlap (see the case study in Figure 7C). For each of the 32 transcripts that are co-regulated *via* multiple dd co-regulation motifs, the miRNAs are in the same miRNA family. Thus, SNPs in the miRNA binding site on the target could exert an impact on the fate of nearly all of its regulations, being lost or gained in a similar ecotype group (see the case study in Figure 7A). In contrast, for 66% of the 65 transcripts co-regulated *via* multiple sd-type co-regulation motifs, the miRNAs are from different families. SNPs in the miRNAs that dynamically regulate the target account for the regulations being lost in different ecotype groups (see the case study in Figure 7B). By taking the climatic variables of ecotypes into account, we found that the co-regulation motifs of a large proportion of the transcripts are newly gained, completely dropped, or collapsed in a list of ecotypes with similar climatic variables (Supplementary Table S10). Taking temperature seasonality as an instance, 82% of the targets in multiple dd co-regulation motifs and 84% of the targets in multiple sd motifs have lower standard deviations of temperature seasonality than those of the whole set of 1,131 ecotypes. For precipitation seasonality, the proportions are 82% of the targets in dd motifs and 90% in the sd motifs. Thus, in spite of the differences in terms of miRNA homology and SNP position between the dd and sd co-regulation motifs, the dynamic regulations toward most of the targets may be reprogrammed in a group of ecotypes exposed to similar ambient temperatures and/or precipitations.

### DD miRNA–miRNA crosstalk regulates more stress-responsive genes than SS and SD miRNA pairs

At the miRNA–miRNA crosstalk level, the DD and SS cooperative miRNA pairs co-regulate more stress-responsive genes than the SD miRNA pairs, and the DD miRNA pairs co-regulate the most stress genes (Figure 6C). Previous studies have detected signatures of positive selection in many stress-responsive genes in plants (Teng et al., 2017; Bondel et al., 2018), which indicates that selective pressure associated with the environmental conditions may have caused the rapid evolution of genes involved in stress responses. Consistently, we observed that SNPs in the miRNA binding sites on the targets account for the dynamic fate of the DD cooperative miRNA pairs across ecotypes, in which more than half of the targets are co-regulated with a dd co-regulation type. Under variable ecological niches, selection largely acts on the stress-responsive genes, which results in the reprograming of the miRNA–miRNA cooperative regulation on these genes, that is two new miRNA–target pairs being gained or both original regulatory pairs being dropped in specific ecotypes.

### SD miRNA–miRNA crosstalk may act as a “transient” bridge in the network

The SD miRNA pairs have a much lower expression correlation than the other types of cooperative miRNA pairs in the network (Figure 6B), reminiscent of the transient and permanent categories of protein–protein interactions. Transient protein interactions are formed only for a short period of time and then broken apart easily (in time), or are tissue- or cell type-specific interactions (in space), whereas permanent protein interactions are maintained through most cellular conditions (Perkins et al., 2010; Greene et al., 2015). Accordingly, permanent protein interactions have a particularly strong relationship with expression, while transient ones do not (Jansen et al., 2002; Bossi and Lehner, 2009). Most transient and permanent protein–protein interactions are important for cellular function (Ghadie and Xia, 2022). The low expression correlation between two miRNAs implies that they may transiently cooperate. Hence, the SD miRNA pairs may play as “transient” bridges between two different subgraph clusters in the network.

We found that our SD miRNA pairs have lower PPImiRFS scores (Meng et al., 2015) than the other two types of miRNA pairs (Figure 6D). The majority of our SD miRNA pairs correspond to the top ranked PPImiRFS scores (i.e., about 50% of SD pairs have the top 21% of PPImiRFS scores), whereas the DD miRNA pairs seldom appear among the top ranks (i.e., 50% pairs have the bottom 5% PPImiRFS scores). For instance, 13 miRNA pairs have the lowest PPImiRFS scores (from 0.07 to 0.37), among which there are 11 SD miRNA pairs. Interestingly, all of the 13 miRNA pairs involve ath-miR5021. ath-miR5021, ath-miR5658, and ath-miR414 have the highest degrees in our network and link with different miRNA families, most of which are with the crosstalk type as SD (Figure 4; Supplementary Table S4). Through these highly-connected miRNAs, the different clusters could communicate each other, demonstrating that these hub miRNAs also act as bottlenecks between the network clusters. We revealed that bottleneck miRNAs tend to be more important mediators for network communication than nonbottlenecks in the miRNA–miRNA crosstalk network (Supplementary Figure S6B). In terms of functional roles, nonhub-bottleneck miRNAs regulate specific functions involved in the regulation of signal transduction and cellular response to gibberellin and alcohol (Supplementary Figure S6C). Hub-bottleneck miRNAs regulate transcripts in various functions (functions of ath-miR5021, ath-miR5658, and ath-miR414 are listed in Supplementary Table S11). ath-miR5021 regulates transcripts involved in DNA recombination and repair, nucleosome assembly, pollination, leaf morphogenesis, regulation of anatomical structure morphogenesis, and glutamine family amino acid catabolic process. KEGG enrichment analysis using clusterProfiler reveals that ath-miR5021 is significantly enriched in the pathway of mismatch repair (adjusted *p*-value <0.05). ath-miR5021 was also identified as a novel miRNA processed in *A. thaliana* sperm cells and pollen (Borges et al., 2011). These support our GO biological annotation of ath-miR5021. Although the SD miRNA–miRNA crosstalk involving ath-miR5021 correspond to the lowest PPImiRFS scores, our study reveals that ath-miR5021 regulates transcripts in various functions and cooperates with different miRNA families, through which these miRNA families could communicate in the network.

### The global expression data based on the Col-0 genome

The expression data used here were produced in the background of reference genome Col-0 (Barciszewska-Pacak et al., 2015; Xu et al., 2018). Different ecotypes may present differential expression of some genes, especially the stress-responsive genes. Differential expression of a major plant stress receptor IRE1 has been detected in *A. thaliana* ecotypes (Afrin et al., 2020). Gene expression of the dehydrins, encoding proteins that help to mitigate the adverse effects of dehydration, differs across ecotypes in Norway spruce, which may be related to climatic variables, such as precipitation, temperature, and day-length (Cepl et al., 2020). In our study, expression profiles were used to assess the quality of the miRNA–miRNA crosstalk network, and compare the expression correlation between miRNA pairs in different groups. Assuming one miRNA is differentially expressed in an ecotype compared with the reference genome, the miRNA regulation is likely to be changed, probably in the form of a new target being gained, an original target being lost, or the target being kept but with a changeable (increased or decreased) binding affinity. It is interesting that the information on differential expression of this miRNA may be reflected by the dynamic fate of its regulations in the ecotypes. Therefore, it is reasonable to infer that expression profiles based on the Col-0 genome are applicable to the analysis in our study.

## Data availability statement

The datasets presented in this study can be found in online repositories. The names of the repository/repositories and accession number(s) can be found in the article/Supplementary material.

## Author contributions

YM and XWu designed the project. XWu constructed networks and wrote the initial manuscript. WC conducted climatic variable analysis. YM, XWu, and XWa analyzed the data. XL, YL, FW, and LL helped with the data analysis. XWu, XWa, WC, XL, and YM revised and edited the manuscript. All authors contributed to the article and approved the submitted version.

## Funding

This work was financially supported by Natural Science Foundation of Zhejiang Province (LY18C050005), National Natural Science Foundation of China (31970637), and Zhejiang Province Public Welfare Technology Application Research Project (LGN18C020005).

## Conflict of interest

The authors declare that the research was conducted in the absence of any commercial or financial relationships that could be construed as a potential conflict of interest.

## Publisher’s note

All claims expressed in this article are solely those of the authors and do not necessarily represent those of their affiliated organizations, or those of the publisher, the editors and the reviewers. Any product that may be evaluated in this article, or claim that may be made by its manufacturer, is not guaranteed or endorsed by the publisher.

## Footnotes

^1^ https://www.arabidopsis.org/

^2^ https://1001genomes.org/data/GMI-MPI/releases/v3.1/

^3^ https://bioinformatics.psb.ugent.be/plaza/versions/plaza_v5_dicots/

^4^ http://bis.zju.edu.cn/PncStress/

## Abbreviations

SNP: Single nucleotide polymorphism
miRNA: microRNA
GO: Gene Ontology
col-0: Columbia-0
ANOVA: Analysis of Variance
coreg: Co-regulation score of two miRNAs toward a target
SS: Both regulatory pairs of a miRNA–miRNA crosstalk being static
DD: Both regulatory pairs of a miRNA–miRNA crosstalk being dynamic
SD: Regulatory pairs of a miRNA–miRNA crosstalk being static and dynamic
